# AFFECT MODERATES THE ASSOCIATION BETWEEN SOCIAL SUPPORT AND RETIREMENT SATISFACTION OVER TIME

**DOI:** 10.1093/geroni/igz038.477

**Published:** 2019-11-08

**Authors:** Kaleena Odd

**Affiliations:** University of Nebraska Omaha, Omaha, Nebraska, United States

## Abstract

Retirement is becoming more important for today’s older adults because they are living longer than before. Recently, research has started to explore how different individual resources (e.g., health or finances) and social resources (e.g., social support or social network size) influence retirement outcomes such as retirement satisfaction. Moreover, the current study sought to examine the influence of time, satisfaction with social support, and affect (i.e., positive or negative) as predictors of retirement satisfaction. Data was obtained from a longitudinal study that explored how older adults in Montreal, Canada adjusted to life in retirement over the course of three years. Hypotheses were tested using a structural equation model that investigated retirement satisfaction as predicted by time, satisfaction with social support, positive affect, and negative affect. Gender differences were also explored. Overall, there was no change over time among the variables. Satisfaction with social support, positive affect, and negative affect were all associated with retirement satisfaction in the expected directions. Positive affect moderated the association between satisfaction with social support and retirement satisfaction, such that the association was stronger for those low in positive affect. Also, negative affect moderated the association between satisfaction with social support and retirement satisfaction as a function of gender. This study extended the literature by exploring how multiple predictors interacted to influence retirement satisfaction over time. Future research should examine how individual and social resources can interact with each other to better understand retirement satisfaction.

